# Abstracts and Gleanings

**Published:** 1882-09-20

**Authors:** 


### ABSTRACTS AND GLEANINGS.

**Mangifera Indica, or Mango.**—The value of this as a therapeutic agent has in my hands proved of so great efficiency that I look upon it as a duty to make known its usefulness to the medical profession. So many new remedies have been introduced within a few years, and so many virtues ascribed to them, that it becomes a delicate subject for a physician to laud a new remedy, for fear that he may be classed as an empiricist. This, however, can not be attributed to me when I tell you that I have no interest in the sale of the article further than the good it will do to suffering humanity, and the benefit the physician who prescribes it will derive. However, I take a certain amount of credit to myself for being the first to introduce it to the medical profession. An excellent description of the article is given in the Supplement to the American Dispensatory.

I have used this drug for upwards of ten years, and can assure that it has never disappointed me in its action. I have experimented with it largely, and have found it an astringent of peculiar power. This bark contains tannin, but it differs from tannic acid in that it will not cause constipation of the bowels. It can be given during active inflammation without the slightest danger. It can be used with great advantage in cholera, typhoid fever, cholera infantum, diarrhœa, and all diseases with liquid discharges from the bowels. My first experiment with this bark was in uterine inflammation and in ulceration of the cervix, by painting the ulcerated surface with the fluid extract (full strength), and using a weak solution as an injection. I have been enabled to overcome the most obstinate diseases of this nature. In gynæcological therapeutics the Mangifera is a most valuable agent. It lessens catamenial pain, cures leucorrhœal discharge, diminishes profuse menstrual evacuation, and corrects menstrual disorders in general. In nasal catarrh a weak solution used with the spray atomizer is, by far, the best agent I have ever used. As an internal remedy for hemorrhage of the uterus, bowels or lungs, and also in muco-purulent discharges from either, I know of no agent equal to it.

In diphtheria this remedy has, perhaps, its largest therapeutic value. I apply it in the form of the fluid extract (full strength) to the fauces with a camel's hair brush, and use a weak solution of it as a gargle. I can honestly assure the medical profession that it has never disappointed me. I do not wish it to be understood that this agent alone will cure all cases of diphtheria, but I do assert that I know of no remedial agent in the whole materia medica that will meet the requirements as well as the Mangifera Indica.

As an antiseptic agent. internally or externally employed, it will prove of great value, as it is decidedly opposed to decomposition.

Prof. Howe says: "I have found the Mangifera of marked service in the treatment of profuse and exhausting menstrual fluxes. In uterine hemorrhage following miscarriage, the agent exerts a powerfully restraining influence upon the hemorrhagic waste. In the sanguineous losses which often occur about the change of life, and when uterine tumors are developing, the Mangifera is the most potent and reliable medicine ever introduced to the notice of the medical profession. I prescribe the fluid extract in five-drop doses every three or four hours. In a short time the influence of the medicine is observed, and in a few days the desired effect is realized. No remedial agent of so great value for the purpose named has been introduced to the profession; it is as near a specific for profuse menstruation and uterine hemorrhage as may be desired. I might report ten or twelve cases in which the medicine exerted just such an action as was wished."

Prof. Moss says of it: "I have tried the fluid extract of Mangifera Indica, and find it to be an astringent of superior power. There is a property in it not found in ordinary astringents. I had a case of chronic diarrhœa of long standing, attended with indigestion, debility, and much pain in the umbilicus. I suspected ulceration, and gave the patient hydrastis and bismuth, but only with partial relief. About this time I secured the Mangifera Indica, put the patient upon it, and cured the diarrhœa. I have waited for several weeks, and find the effect so far permanent. I am much pleased with it, and predict that it will prove a valuable acquisition to our list of astringents."

It has the following advantages over other astringents: The dose is small, easily taken, has no disagreeable taste, does not derange the stomach; hence, it is well adapted to infants and persons with fastidious stomachs. It is rapid in its action, and more certain in its effects than most other remedies.

For the benefit of humanity, I introduce this remedial agent to the medical profession, hoping that my brethren will try and prove its value from their own standpoint.—*Eclectic Medical Journal.*

**Surgery and the Doctrine of Evolution.**—C. Pittfield Mitchell, M. R. C. S., of Orange, N. J., contributes to the "New York Medical Journal and Obstetrical Review" for September, 1882, an essay, entitled, "An Evolution Aspect of the Healing of Wounds, with Deductions as to Treatment." As the author tells us in a prefatory statement, this is an application of the Spencerian doctrine of evolution to some of the phenomena of reparative action. The essay sets out with a classification of methods of repair from the standpoint adopted. Next, the grounds for this classification are given, and incidentally we are introduced to an important conception—arguing that, since whatever is profitable to an organism, in the way of structural or functional variation, will be taken advantage of by heredity and natural selection, the functional changes naturally involved in recovery from disease will come within the sphere of their operations. With the zymotic diseases, for instance, natural selection may segregate, and heredity may fix, both the physiological peculiarity which insures immunity, and the physiological activities which establish the *status quo* when the disease has been contracted. Entering upon the immediate topic of the paper, the phenomena elicited by an incised wound, as the occlusion of arteries, the organization of plastic lymph, the development of granulations, and the physiological adjustment of the tissues to new external conditions, are viewed as non-specific functions of the tissues injured superadded to their specific functions. Deducing the evolution of these phenomena from the known action of physical forces, the shares taken by natural selection and sexual selection as factors are then dwelt upon. Special attention is directed to the protective value of the plastic lymph forming on the surfaces of wounds, and the evolutional steps are described by which this function is acquired. Passing from the structural, the evolution of certain more strictly functional adaptations is next considered. Knowing, in general terms, the atmospheric and other forces to which wounded tissues in the past must have been exposed, the corresponding accommodations of function are inferred. Thus, the general conclusion is drawn that "the molecular constitution of wounded tissues should fit them, on the average, for contact with a mean atmosphere, and certain moderate deviations from this mean." It is pointed out that, although traumatic injuries are not necessary accompaniments of life, they are of such frequency among the lower animals and man as to give validity to this conclusion. An absence of organized adjustments of function to the remaining forces commonly incident upon wounds is inferred from the inconstancy, diversity and nature of these forces. Such deductions are shown to harmonize with experience, and certain principles of treatment for healthy wounds are presented as corollaries. The gist of them is, that, so far as the exigencies of practice will permit, wounds should be shielded from the incidence of any force to which we may know *a priori* there cannot exist an organized adaptation. A normal atmosphere should be maintained, and cleanliness shonld be absolute at every step. Believing that the plasma exuding from the severed tissues is, by "its chemical and mechanical properties, and contact with environing forces during evolutionary time, specially fitted to protect the less stable cells which lie underneath," much importance is attached to the preservation of its integrity. "Wounds should remain open until the surfaces have become glazed, and all interfering applications should be scrupulously withheld." Finally, a verification of these inferences is found in the facts disclosed by Dr. McVail, in his paper in the "British Medical Journal," for July 23, 1882, on the results of "Ten Years' Surgery in Kilmarnock Infirmary." The method of dressing employed (dry-dressing) essentially fulfilled the above-mentioned theoretical requirements, and gave, on comparative analysis, the "best general results covering a lengthened period of time that have ever been recorded in the history of British hospital surgery;" and the last group of cases reported—numbering 421, including 90 operations, 23 major amputations, 45 injuries, 52 abscesses and 7 compound fractures—showed not a single fatality from any cause.

**Colostrum in the Milk as a cause of Cholera Infantum.**—Dr. Gilbert, in Louisville Medical News, says: "Another common cause of intestinal irritation in infants, which of course comes under the general head of "bad food," is the presence of colostrum in the mother's milk. There are various conditions of the mother under which this substance—which is not only indigestible, but a decided irritant—may make its appearance in the milk, viz.: menstruation, pregnancy, mental anxiety, etc.; but a condition which, under my observation, has caused the appearance of large quantities of colostrum in the milk is prolonged sexual excitement; and, so far as I am aware, attention has not heretofore been called to this fact. I will report a few cases from some notes in my memorandum-book.

Two summers ago Mrs. B., of Nashville, visited this city, bringing with her a healthy infant six months of age. After about three weeks time her husband visited her, and on the morning following his arrival their child was attacked with a severe serous diarrhœa, complicated with convulsions, which terminated in death in thirty-six hours. On inquiry the mother admitted that at three successive intervals during the night she and her husband had indulged in sexual intercourse, and that the child had been put to the breast immediately after each act.

Another case was that of an infant wet-nursed by a mulatto woman for a delicate lady. The nurse had remained at the lady's house, day and night, caring for the infant exclusively for a period of five weeks, during which time the child remained in perfect health. The husband of the nurse having returned from a protracted trip to New Orleans as porter on a steamboat, she was permitted to go home and spend the night with him, upon promise to return early next morning; which promise she kept, returning fagged and weary (as the lady described her), and immediately gave the breast to the infant. In an hour or two thereafter this infant was seized with severe symptoms of cholera infantum. I was asked to see the child, and, after hearing the history of the case, at once suspected colostrum in the nurse's breast, consequent upon a night of venery, which the nurse admitted on interrogation. The microscope showed an abundance of colostrum in a sample of her milk obtained as late as 9 o'clock upon this day.

I will mention still another case occurring during the heated term of last month (June, 1882.) The husband (a stout laboring man) of a healthy Irish woman with an infant ten months old, had been absent about twenty days, but returned early on one Sunday morning. During the day and evening he had sexual intercourse several times with his wife, and the infant was put to the breast and allowed to nurse freely during the intervals between the sexual acts. Two hours before daylight on the following Monday morning I saw the child presenting a typical case of cholera infantum, which came very near terminating fatally.

I could report a number of other cases similar to the above, but believe these sufficient to suggest, if they do not demonstrate, this theory of causation.

In conclusion, I would suggest that while we are busy in correcting the various conditions which have hitherto been admitted as causes of cholera infantum, it would be well to caution the nursing mother against protracted sexual excitement, and especially against putting her infant to the breast very soon after such indulgence; possibly in three or four hours after sexual intercourse the milk may regain its normal condition. We should not lose sight of the fact that during dentition the physiological process known as development of the gastro-intestinal follicles is also taking place, which causes a great determination of blood to the parts, thus rendering the intestinal mucous membrane hypersensitive and more easily deranged by any indigestible substance in the infant's food, no matter how produced."

**Pasteur's Investigations on Rabies.**—The Paris correspondent of the British Medical writes of M. Pasteur:

This eminent biologist has made some most important contributions to science, and his name will ever be connected with his ingenious researches on fermentation, and other important discoveries; but he has drifted into a more speculative kind of scientific experiments. As an example of this may be mentioned his recent experiments with the saliva from the mouth of a child with rabies, with which he innoculated rabbits and guinea-pigs. All the animals died, and their blood was found to contain myriads of micro-organisms, which he concluded to be the specific germs that produce hydrophobia. He then performed a second series of experiments, by innoculating other rabbits with the blood of those that had succumbed from the first innoculation. These also died, and their blood was found to contain the same micro-organisms. He, however, soon discovered by further experiments, but this time with the saliva of children who had died from other diseases, that the results were precisely similar to those observed with the saliva of the child. In pushing his experiments still further, but with the saliva of a healthy adult, he met with the same results and the same germs as in the preceding cases. This rather puzzled the persevering experimenter, but he is not so easily beaten; and if he has not yet discovered the real nature of the virus of rabies, he fancies he has laid his hand on the organ that secretes it. According to him, the virus of the rabies is not secreted by the salivary glands, but by the brain—or rather, the latter is the seat of the malady; and, in support of his thesis, he innoculated a small portion of the bulbous extremity of the medulla oblongata of a rabid animal under the cerebral covering of a healthy animal. The latter became rabid. These results were recently communicated to the Academy of Medicine, in a paper read by the general secretary for the learned experimenter, which called forth some trenchant remarks from M. Bechamp, who positively refused to accept the principle on which M. Pasteur has hitherto founded most of his theories, and added that it is not outside the body that one must look for the germs or elements of destruction; but they are to be found in our body, in the form of microzymes, which are the only cause of all fermentation, and the lowest element to which our organism can be reduced. Nothing daunted, however, M. Pasteur continues his parasitic warfare with unbroken zeal; and, by further experiments with human saliva, he has made the startling discovery that the saliva of a person fasting is venomous, as it contains the same parasites as those found in the saliva of children above described; but that, on the person breaking his fast, his saliva is deprived of the venomous quality, as the parasites are taken into the stomach with the food. All this is terrible to contemplate; and even M. Pasteur was confounded, as the result of his experiment was as awful as it was unexpected. The learned biologist made no attempt to offer any explanation, but said that he would for the present only point to the fact, which, he added, was in itself very suggestive.—*Med. ard Surg. Rep.*

**Treatment of Snake-bite.**—Dr. Vincent Richards (Indian Medical Gazette, January) offers the following suggestions as to the treatment of snake-bite: 1. In the case of the bite being on a limb, a ligature should be at once applied above the bitten part, care being taken that it is sufficiently tight to prevent any blood being taken up into the general circulation from the distal end; give a full dose of opium (forty minims of the tincture, or half a grain of morphia) hypodermically. 2. Inject hypodermically, into the bitten part, a solution of the permanganate of potash (one grain to a drachm),and well press the part with the fingers. 3. Open a vein below the bitten part, and wind round the limb an elastic bandage, so as to exsanguinate the limb below the bitten part. 4. Cut through the bitten part, and, when dry, apply pulverized permanganate, and then loosen the ligature. In the case of a person bitten on the trunk, any treatment, however prompt, may be useless; but it would be well to inject the part with permanganate, and give a full dose of opium. "It may not be generally known to the members of the profession that a poisonous bite may be easily ascertained by cutting through the punctures into the areolar tissue beneath, when, if a red-currant-jelly-like appearance be observable, the bite is poisonous. The merit of pointing out the diagnostic value of this local appearance is due to Dr. Wall."—*Med. and Surg. Rep.*

**A Dose of Quicksilver.**—One night at about one o'clock, the writer was summoned to visit a patient, who, the messenger reported, had been sick ten or twelve days, and, as he seemed to be quite dangerously ill, requested immediate attendance. He had been attended by two physicians of considerable note, who diagnosticated the case as one of intussusception, and, after exhausting all other means within their knowledge, resorted to the final one of administering about two tablespoonsful of quicksilver. After watching the opposite orifice for the appearance of the lively fluid about three hours, without realizing their hopes and expectations, they gave it up as a bad job, and pronounced sentence of death on the poor victim; for, they informed the friends, if he did not die from the effects of the disease, he certainly would from the medicine, and they left instructions to make him as comfortable as possible as long as he lived, for they could do no more. We found the patient a man of about 65 years of age, tall and spare, suffering great pain all over the abdomen, which was very tender to the slightest touch and considerably distended; the knees were drawn up; there was an anxious expression of countenance, a slow, feeble pulse, cold perspiration and a coated tongue. The case had the general appearance of one suffering from peritonitis and we so treated it. Instead of using cathartics to force the contents through the paralyzed intestines, the bowels were kept perfectly quiet by the free use of opium for about seven days; arsenic, bryonia and aconite were used internally with hot fomentations of hops applied externally. At the end of a week nearly all traces of the inflammation had disappeared, when a decoction of senna and aloes was used with directions to retain it as long as possible; after a few hours the bowels began to move freely, and the two first evacuations contained about a tablespoonful of pure quicksilver and about three times that amount of what appeared to be a black oxide of mercury.

After this the patient went on to a rapid recovery without the slightest indications of ptyalism or any other symptoms of mercurial poisoning. It has generally been supposed that if quicksilver does not produce the desired cathartic effect in a short time, it will cause death; but this case seems to refute the popular idea. If gangrene has commenced in an intestine in a case of intussusception before or soon after the administration of the quicksilver, the latter, no doubt, would hasten the fatal moment by rupturing the intestine; but, as the above case shows, no immediately fatal results follow when the intestines are strong enough to hold it. We believe that it should never be given under any circumstances, for it never did good.—*Investigator.*

**Salicylate of Potassa in Acute Rheumatism and Dyspepsia.**—Dr. M. Donnelly, of New York City, in Virginia Medical Monthly, said of the above remedy at the American Medical Association: "I was convinced that there was merit in salicylic acid, provided it could be employed with safety, and I made some experiments, hoping to find some alkali in greater proportion than soda, so as to produce a thoroughly alkaline salicylate, which I finally found in the bicarbonate of potash.

Two parts of bicarbonate of potash and one part of salicylic acid dissolved in a little water, formed a neutral solution. The potash was then increased in quantity until one part of the acid united with two parts of potash—say ten grains of acid to twenty grains of alkali in a drachm of water—formed a clear alkaline solution. This solution evaporated to dryness, left a strong alkaline salt of grayish color, sweetish taste, soluble in double its weight of water, which I called salicylate of potassa. The action of this remedy is very rapid. It becomes absorbed rapidly, and its influence is felt in a few hours in mitigation of pain. In mild cases the urine and perspiration become alkaline in character in a few hours, but in severe cases several days are required to effect these secretions. This point once reached, improvement is progressive. The sediment in the urine disappears, the metastatic character of rheumatism goes with it, and the case goes on to recovery. The remedy is used until all pain and swelling are relieved, and it is then necessary to guard against relapses, which appear at this stage, owing to the lessened powers of resistance to cold of the patient, caused by thinness of the blood. To establish the rich, warm, normal condition of the blood is most readily accomplished by the use of an alkaline form of iron, and the best of all is tartrate of iron and potassa. As to the causes of rheumatism, most all physicians agree that abnormal digestive secretions take a prominent part in forming the lactic acid in the blood.

This remedy is too valuable in the treatment of flatulence, pyrosis, heartburn and loss of appetite—in fact, all symptoms of dyspepsia of the acid form—to be passed without mention. Its power in controlling fermentation first led me to prescribe it in flatulence, given in powder after meals. It not only relieved this symptom, but digestion improved under its use. With an experience of over two hundred cases of dyspepsia cured by salicylate of potassa, I can unhesitatingly recommend it for any of the bitter tonics. It will be found successful in nine cases out of ten, the tenth one requiring mineral acids, owing to the bilious condition of the patient.

**The Secrets of our Patients**.—British Medical Journal, March 18, 1882: The Journal de Medicine de Paris contains a letter from a correspondent detailing a hypothetical case, in which the medical attendant delivers a woman of a syphilitic child, when, to his knowledge, the father is exempt from the disease, and desires to know whether it is the duty of the medical attendant to inform the father of the nature of the disease from which the child is suffering. M. Diday replies to the latter, and maintains that such a case offers no exception to the general rule, that the secrets of our patients are inviolable. He points out that when the child is born dead, as a rule, no questions would be asked, and if they were it would be sufficient to say that there was commencing putrefaction; but when syphilitic symptoms are manifested by the child after birth, he thinks the medical man can easily discover the real state of things; and he believes that it is only necessary for him to insist on the mother nursing the child herself, so as to avoid infecting anyone else; and should she herself show any symptoms of the disease, to submit herself at once to treatment, and to persevere in it actively and to the end. We may add that we quite agree with M. Diday; any other view is obviously founded on a principle which would make one law for the husband and another for the wife;. for who ever heard of a medical man feeling himself bound to tell a wife that her husband had acquired syphilis?—*Mich. Med. News.*

**Does Quinia Increase or Lessen Intracranial Blood Supply?**—Those who have given it in small doses, say 2 to 5 grains every four hours, have seen the pulse increase in force and frequency, the conjunctivæ injected and retinal vessels full and the activity of the functions generally raised above the usual level. They hold, therefore, that quinia increases the intracranial circulation. Experimental evidence has also been adduced. The meninges of animals exposed, the membranes are seen under the action of quinia to become more vascular and the cephale-hæmadynometer registers higher intra-cranial blood-pressure. The very opposite conditions are observed when large doses are administered, say ʒi or more. Then the pulse is slowed, the face grows pale, the retinal vessels become small and there is much of that tinnitus and vertigo significant of cerebral anæmia. Experimental researches show that the cerebral meninges are pale, exsanguine, and blood-pressure low. Still more conclusive is that accidental experiment made on man—the quinia amaurosis—in which there is an extreme pallor of the optic discs, the vessels of the largest size appearing as minute threads, and large numbers of vessels usually in view have disappeared. Several examples of this kind have been observed by Knapp and other ophthalmologists after the exhibition of large doses of quinia.—*Bartholow, Med. News, Aug. 26.*

**The Anti Fat Dietary.**—Our corpulent friends may be interested in the report of Mr. Joseph Harrass's attempts to get rid of his superfluous burden of flesh, especially as the dietary followed does not seem, on the face of it, to be an objectionable one, and has not proved injurious to health in his case. The facts are stated in the Herald of Health as follows:

He was corpulent, had irregular and feeble action of the heart, tendency to fainting, difficulty of breathing, and many disagreeable sensations in the head indicative of nervous exhaustion. Height, five feet six inches; normal weight, one hundred and fifty pounds; age, fifty-nine; weight at beginning of treatment, two hundred pounds. Began treatment October 8th. Treatment as follows: Breakfast—Vegetables, brown bread (toasted), water; with lemon juice, and occasionally oatmeal. Dinner—Vegetables, brown bread, water and plain pudding. Supper—Brown bread (toasted), stewed fruit and water. No tea, coffee, cocoa or milk, except skimmed, and only a trifle of butter. Result:

| | | |
|---|---|---|
| End of October weighed | | 187 ℔s. |
| " November | " | 182 " |
| " December | " | 177 " |
| " January | " | 174 " |
| " February | " | 173 " |
| " March | " | 170 " |
| " April | " | 168 " |
| " May | " | 166 " |
| " June | " | 186 " |
| Present weight | | 150 " |

All the distressing symptoms have been relieved, and the patient is so well he can again carry on his business. His physical and mental strength have been greatly increased.

Mr. Harrass says he has suffered no serious discomfort from his diet, except when away from home, and he feels as if he had learned an important lesson as to how to reduce his corpulence—which has been such a source of discomfort—and once more enjoy life.—*Boston Jour. of Chem.*

**Intermittent Fever and Quinine Hypodermically.**—In the Lancet Dr. P. A. Smith contributes the following case: A sailor, aged 32, had been sick for three months, with continuous quotidian fever and scurvy. On the day after his admission into the hospital it was deemed advisable to test the statements he had made as to his febrile condition, by allowing him to remain in bed and giving no medicine. At 8:45 a. m. he felt cold, and at 9 a. m. his temperature in the axilla was 102° F., and at 11 a. m. 104°; after which there was a gradual decrease every half hour until 1 a. m. when it was 100.4 F. He was ordered a hypodermic injection of six grains of quinine at 8 p. m., to be repeated at 8 the following morning. It was reported the next day that he passed freely and without fever through the usual times of having the paroxysms. There was no increase of temperature and no uneasiness, and he says this is the first time he has been free from fever for the past three months. He had two injections at the same hours on the two following days, when it was noticed that the arm where the injection was given was somewhat tender, and the quinine was given by the mouth, dissolved in tartaric acid, which mixture was continued in diminishing doses for the next fourteen days. He daily gained in strength, and left, three weeks after admission, in excellent spirits. No slough or abscess marked the site of the injections, and the pain experienced at the time of injection was slight and soon passed away.—*Med. and Surg. Rep.*

**Immunity of the Chinese from Disease.**—The medical officer of the State Board of Health of San Francisco has given his testimony as to the effects of residence among the Chinese, which has been laid before the Congress. He states that he never knew any disease or pestilence originating or spreading in the Chinese quarter. He admits that the Chinese live quite close, and attributes their healthy condition and immunity from disease to their frugal life. "They eat," he says, "only what is necessary to live upon. They eat to live, and do not live to eat. They are clean in their habits, and they drink no whisky. I have never seen a drunken Chinaman in my life. They consequently obtain a better resisting power to the attack of disease. They constantly wash themselves, and keep themselves and their clothes clean. The death-rate is greater among the whites than among the Chinese—greater with adult white people than with adult Chinamen. There have been no epidemics among them, and there has been less small-pox among them than among the whites, the ratio of population being allowed."—*Boston Jour. of Chem.*

**Carbolized Nerves as Ligatures.**—Dr. Jno. A. Wyeth, of New York city, publishes in the Archives of Medicine for June an account of certain experiments which he has made with carbolized nerves as ligatures. The advantages presented by nerve-tissue for this purpose are, that it is easily obtained in a perfectly fresh state from the butcher, is strong by virtue of its neurilemma, and soft, because each nerve-fiber is surrounded by the cushion-like white substance of Schwann. He tied the carotid in a horse and also in a greyhound with the carbolized nerves, and found on subsequent dissection (in the fifth week after the operations) that, while the artery was completely occluded, its continuity was unbroken, there being only a depressed ring, scarcely perceptible, at the point where the ligature constricted it. The ligature, moreover, had undergone complete absorption. The superior strength and smoothness of nerve-tissue seem to bespeak for it great advantages over the animal ligatures now in use.—*Louisville Med. News.*

**Castor Oil as a Lactigogue.**—In the Transactions of the Lancaster County (Pa.) Medical Society, Dr. P. J. Roebuck reports the following: Mrs. O., aged 42 years, has given birth to nine children. The first seven were raised by artificial food. I attended the woman in her eighth confinement, August 8th, 1878, and determined on an effort to establish the lacteal secretion. The treatment consisted in a liberal diet from the outset, such as eggs, oysters, beef, milk, cream, etc., with a thorough and continued application of castor oil to the mammary gland. With this treatment I succeeded fully, and enabled the mother to nurse her child for the first time in eight confinements, for a period of eighteen months. Jan. 16th of the present year the woman gave birth to her ninth child, and is now, under the same treatment, supplying all the milk necessary to the comfort and growth of her child.—*Med. Summary.*

**The Value of Viscum Album or Mistletoe in Affcctions of the Heart.**—In a communication to the Practitioner for November, 1881, Dr. R. Park, of Glasgow, states that among a clientele consisting mostly of miners, he found an unusually large proportion of cases of heart disease. Some were displacements, some valvular disease, some hypertrophy, some asthenia and palpitation simply. Rheumatism and the cramped position in which the men worked were variously blamed for the morbid conditions. Whatever the exact pathological condition might be, incompetency and tumultuous distressing cardiac action were the immediate symptoms calling for treatment in those that presented themselves. For these he prescribed ʒ ss doses of tincture of mistletoe every four hours, with the very best results; all the patients returning with the above symptoms ameliorated. The Doctor regards the mistletoe as a valuable substitute for digitalis.—*Med. and Surg. Rep.*

**Large Dose of Chloral.**—The Chicago Medical Review reports a case where a patient with epileptic mania took one ounce of chloral hydrate. After some time the stomach-pump was resorted to, and hypodermic injections of whisky and strychnia were administered. The patient sank in a few minutes into a deep slumber which lasted forty-eight hours. On the third day a vivid scarlatiniform eruption appeared over the whole body, which fully desquamated within two days. Otherwise no unpleasant results followed except marked tenderness, for a long time, of the buccal mucous membrane. The fits were less frequent after the accident.—*Maryland Med. Jour.*

